# Human coronavirus NL63 nsp1 induces degradation of RNA polymerase II to inhibit host protein synthesis

**DOI:** 10.1371/journal.ppat.1012329

**Published:** 2024-06-20

**Authors:** Kala Hardy, Michael Lutz, Toru Takimoto

**Affiliations:** Department of Microbiology and Immunology, University of Rochester Medical Center, Rochester, New York, United States of America; Cleveland Clinic Florida, UNITED STATES

## Abstract

Coronavirus (CoV) nonstructural protein 1 (nsp1) is considered a pathogenic factor due to its ability to inhibit host antiviral responses by inducing general shutoff of host protein synthesis. Nsp1 is expressed by α- and β-CoVs, but its functions and strategies to induce host shutoff are not fully elucidated. We compared the nsp1s from two β-CoVs (SARS-CoV and SARS-CoV-2) and two α-CoVs (NL63 and 229E) and found that NL63 nsp1 has the strongest shutoff activity. Unlike SARS-CoV nsp1s, which bind to 40S ribosomes and block translation of cellular mRNA, NL63 nsp1 did not inhibit translation of mRNAs transfected into cells. Instead, NL63 nsp1 localized to the nucleus and specifically inhibited transcription of genes under an RNA polymerase II (RNAPII) promoter. Further analysis revealed that NL63 nsp1 induces degradation of the largest subunit of RNAPII, Rpb1. This degradation was detected regardless of the phosphorylation state of Rpb1 and was blocked by the proteasome inhibitor MG132. We also found that Rpb1 was ubiquitinated in NL63-infected cells, and inhibition of ubiquitination by a ubiquitin activating enzyme inhibitor (TAK243) prevented degradation of Rpb1 in virus-infected cells. These data reveal an unrecognized strategy of host shutoff by human α-CoV NL63: targeting host transcription by inducing Rpb1 degradation to prevent host protein expression. Our study indicates that viruses within the same family can use completely distinct mechanisms to regulate host antiviral responses.

## Author summary

Viruses use a variety of mechanisms to prevent host antiviral responses, including inhibiting host transcription or translation. α- and β-coronaviruses express nsp1, which induces general host shutoff to block antiviral responses. Studies of SARS-CoV-2 nsp1 showed that it inhibits host mRNA translation by blocking the mRNA entry site of 40S ribosomes though its C-terminal domain (CTD). However, human α-coronavirus nsp1s lack this CTD, so how they induce shutoff is not known. In this study, we show that human α-coronavirus NL63 uses its nsp1 protein to induce ubiquitination and subsequent degradation of host RNA polymerase II, thus preventing global transcription of host mRNA. Our study is the first to show that a CoV nsp1 induces host shutoff by targeting and degrading RNAPII. This finding indicates that viruses within the same family can take completely different approaches to regulate host antiviral responses though the same protein. This study highlights the variation in viral approaches to regulate host antiviral responses.

## Introduction

Virus infection triggers a wide range of host defense machinery, such as innate immune responses and inflammation. Many viruses express proteins to counteract host antiviral activity and immune responses. One of the major viral strategies to regulate host responses is to induce general shutoff of host protein synthesis. This is observed in many viruses, including respiratory RNA viruses such as influenza A virus and coronaviruses (CoVs) [1–4]. CoVs represent a large family of RNA viruses that infect various animals and humans. They occasionally cross species barriers to cause epidemics or pandemics. So far, seven CoVs are known to infect humans. Two alpha- (α-) CoVs (NL63 and 229E) and two beta- (β-) CoVs (OC43 and HKU1) cause mild respiratory infection and circulate continuously in human populations [5,6]. Severe Acute Respiratory Syndrome CoVs (SARS-CoV-1 and -2) and Middle East Respiratory Syndrome CoV (MERS-CoV), which belong to the β-CoV genus, cause more serious respiratory diseases [7–9]. Both α- and β-CoVs express nonstructural protein 1 (nsp1), which is known to control antiviral responses by globally reducing host protein expression [4]. It is considered to be a major virulence factor since nsp1-deletion mutants of SARS-CoV-1 are highly attenuated in mice [10]. Interestingly, nsp1 is one of the least conserved nonstructural proteins in CoVs. Although the sequence is not highly conserved, the N-terminal globular core of CoV nsp1 proteins show structural similarities. They all contain six antiparallel β-strands and two α-helices [11–15]. The six β-strands that form a β-barrel fold are considered as a common fold in α- and β-CoV nsp1. Nsp1 of human α-CoVs NL63 and 229E is composed of 110 amino acids, while β-CoV nsp1s have an additional C-terminal domain (CTD). This CTD varies in size between the four lineages [4]. Importantly, studies of nsp1 from both SARS-CoVs indicate that these nsp1s use their CTD to inhibit translation by blocking the mRNA entry site of 40S ribosomal subunits [16–18]. The two helices at the CTD stabilize each other and its shape completely blocks the regular mRNA path. Although blocking translation is its primary approach to induce host shutoff, SARS-CoV nsp1s have also been shown to induce mRNA degradation [19–21] and disrupt mRNA export machinery [22].

In contrast to the studies on β-CoV nsp1s, little is known about the mechanism of shutoff induced by α-CoV nsp1. Importantly, α-CoV nsp1s lack the CTD which plays a key role in β-CoV host shutoff. Here, we report that nsp1 from human α-CoV NL63 showed the strongest shutoff activity as determined by a reporter gene assay. Further analysis revealed that NL63 nsp1 takes a completely different approach from other CoVs to induce host shutoff. NL63 nsp1 is translocated to the nucleus and induces ubiquitination and degradation of host RNA polymerase II (RNAPII) to reduce host transcription. This is the first report that shows that a human CoV targets host RNAPII to induce shutoff and block host antiviral responses.

## Results

### Phenotypic differences between human CoV nsp1s

**Fig 1:**
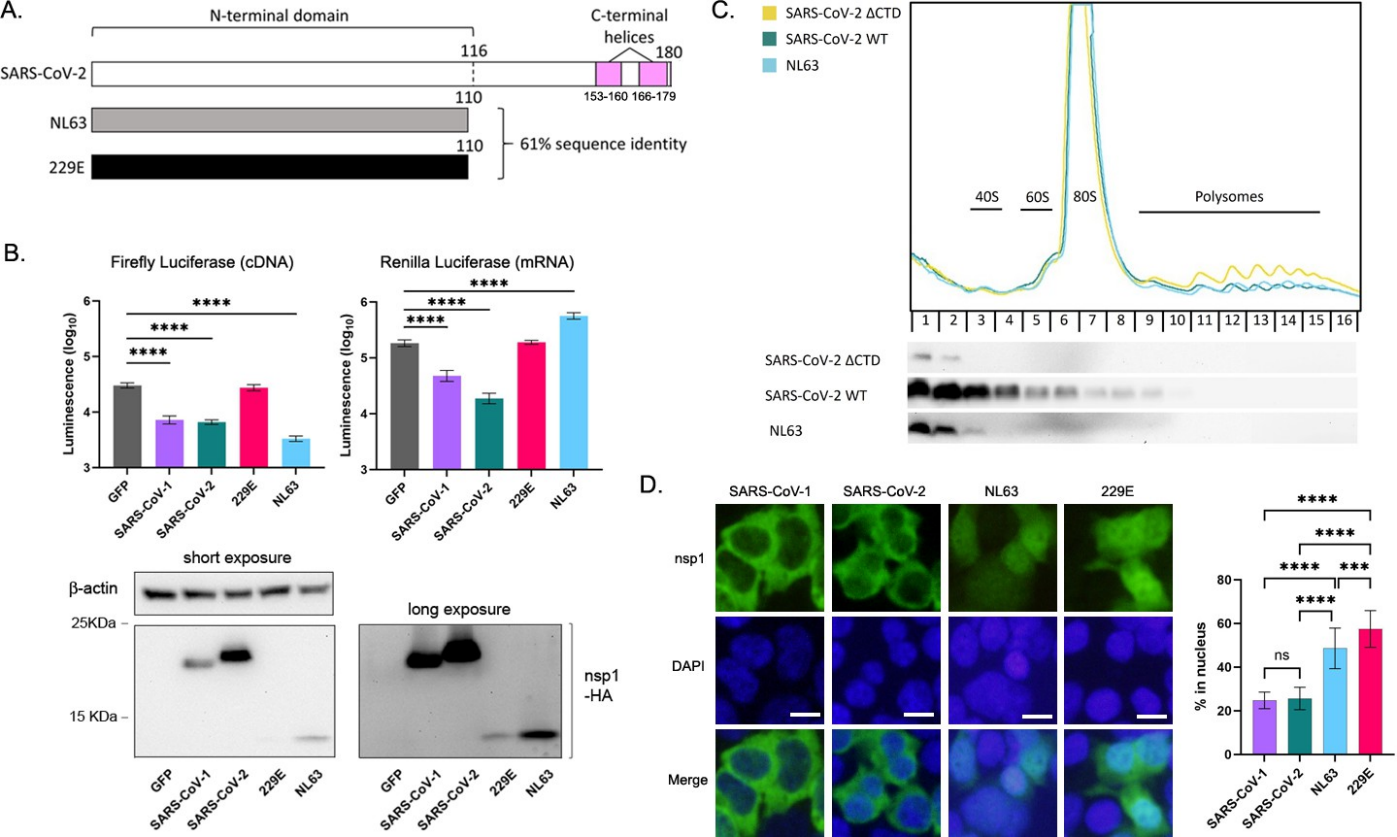
Phenotypic differences between human CoV nsp1s. (A) Schematic diagram comparing nsp1 from SARS-CoV-2 to nsp1 from NL63 and 229E. The number of amino acids in each protein is indicated, and the N-terminal domain and C-terminal helices of SARS-CoV-2 nsp1 are labeled. (B, top) 293T cells were transfected with pCAGGS-FF-Luc, *in vitro* synthesized Ren-Luc mRNAs, and *in vitro* synthesized mRNAs for GFP or the indicated nsp1 for 16 h. Luciferase activity was measured for both firefly luciferase (top left) and renilla luciferase (top right). The mean and standard deviation are shown. Ordinary one-way ANOVA with Dunnett’s multiple comparisons test. ****, p < 0.0001. N = 3. (B, bottom) Nsp1 expression in cell lysate used for the luciferase assay was determined by Western blot using anti-HA Ab. Anti-β-actin Ab was used for a loading control. (C) 293T cells were transfected with the indicated nsp1 cDNAs. Twenty-four h post transfection, cells were treated with CHX and lysed. Lysate was layered over a linear sucrose gradient, centrifuged, and fractions were collected. Top, absorbance at 254 nm measured during fractionation. Bottom, proteins were precipitated from each fraction and analyzed by western blot using anti-HA Ab. (D) 293T cells transfected with mRNA expressing the indicated nsp1-HA were stained for IF with anti-HA Ab. Cell nuclei were counterstained with DAPI. Images were captured using the 40x objective. Bar indicates 20 μm. The signals in total and nuclear regions were quantified by Image J and the percentage of nsp1 in the nucleus was calculated. Ordinary one-way ANOVA with Tukey’s multiple comparisons test. ****, p < 0.0001. ***, p < 0.001. ns, not significant. N = 3.

All human CoVs express nsp1, which is considered to play a significant role in regulating host immune responses. However, differences in nsp1 activity and mechanism of action in various CoVs have not been fully elucidated. Because α-CoV nsp1s lack the domain known to be required for host shutoff by SARS-CoV nsp1s (Fig 1A), we first compared the shutoff activity of two β-CoVs (SARS-CoV-1 and -2) and two α-CoVs (NL63 and 229E). The amino acid sequences of the nsp1s used are provided in S1 Fig. To elucidate which stage of host protein expression nsp1 disrupts, we transfected 293T cells with *in vitro* transcribed capped and polyadenylated mRNA encoding HA-tagged nsp1 along with two types of luciferase: plasmid DNA encoding firefly luciferase (FF-Luc) and mRNA encoding renilla luciferase (Ren-Luc). We then measured the luciferase activities. Nsp1 from SARS-CoV-1 and -2 reduced the expression of both FF- and Ren-Luc, as is expected for a host shutoff protein that inhibits translation (Fig 1B). The α-CoV 229E nsp1 did not show shutoff activity in this assay, while NL63 nsp1 demonstrated the strongest inhibition of FF-Luc expression from cDNA, even with less nsp1 expression compared to SARS-CoVs (Fig 1B). Interestingly, NL63 nsp1 did not reduce the expression of Ren-Luc from mRNA. Instead, it slightly enhanced the expression of Ren-Luc. This could be due to transfected mRNAs having better access to ribosomes if cellular mRNAs are shut down prior to reaching ribosomes. These results indicate variation in nsp1 shutoff activities among the CoVs and that NL63 nsp1 does not block translation of capped and polyadenylated mRNAs transfected into cells.

Because NL63 nsp1 did not inhibit translation of FF-Luc mRNAs, unlike SARS-CoV-1 or -2 nsp1, we used a polysome fractionation assay to determine whether NL63 nsp1 binds to ribosomes, as has been reported for SARS-CoV nsp1s. Consistent with other reports [18], SARS-CoV-2 nsp1 was recovered from fractions containing 80S ribosomes and ribosomal subunits (Fig 1C). In contrast, NL63 nsp1 was not detected in ribosome-containing fractions, similar to SARS-CoV-2 nsp1 lacking the CTD (ΔCTD), which was previously characterized to lack ribosome-binding activity. We next analyzed the localization of nsp1s in 293T cells by immunofluorescence (IF) staining. Consistent with previous reports [20], nsp1 from the SARS-CoVs predominantly localized in the cytoplasm (Fig 1D). In contrast, NL63 nsp1 was found in both the nucleus and the cytoplasm in 293T cells, suggesting a function in the nucleus to induce host shutoff.

### NL63 nsp1 inhibits transcription by RNA polymerase II

**Fig 2:**
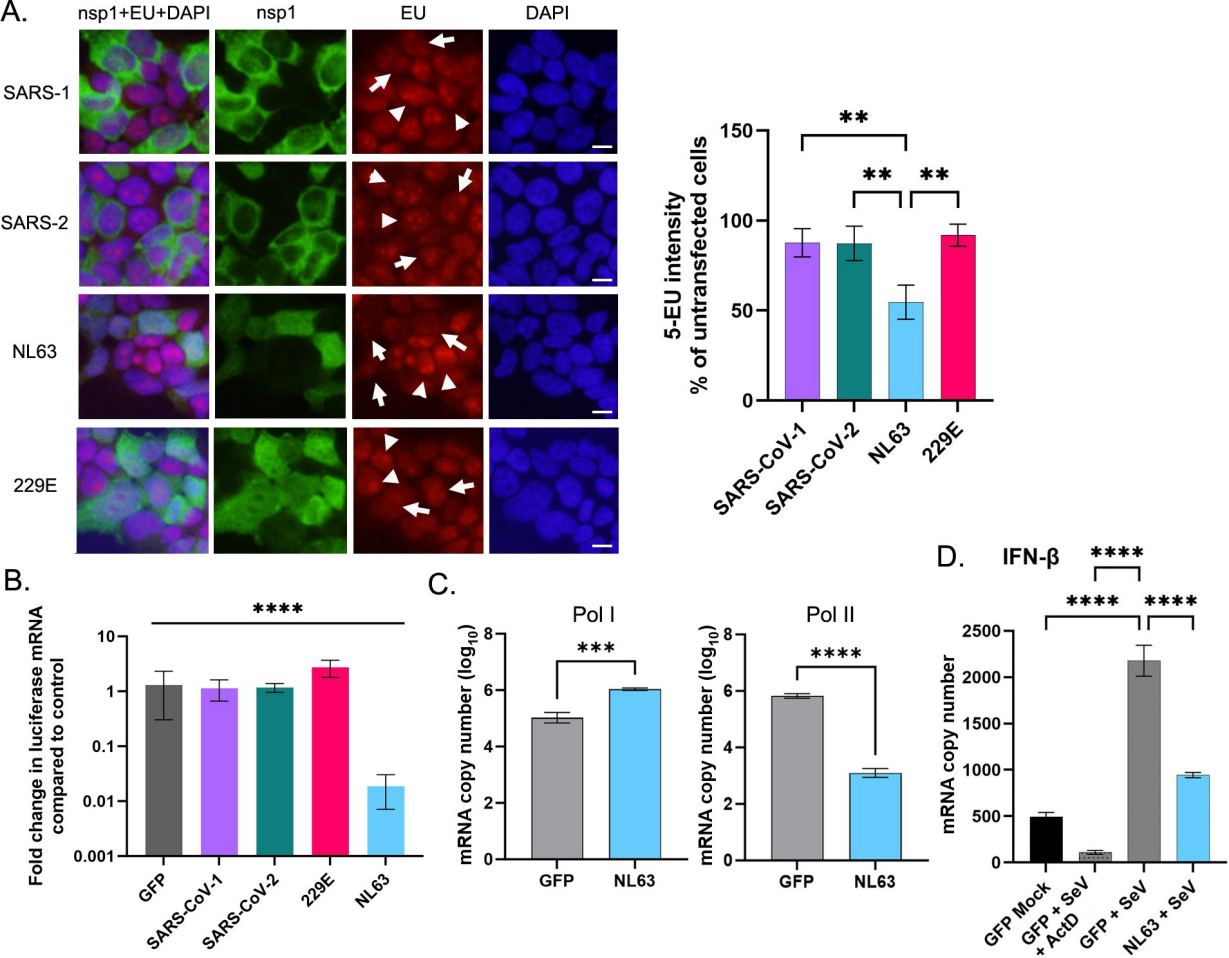
NL63 nsp1 inhibits transcription by RNA polymerase II. (A) 293T cells were transfected with *in vitro* synthesized mRNAs encoding nsp1 for 6 h and labeled with 5-EU for 1 h. Fixed cells were processed for Click-it reaction with Alexa Fluor 594 conjugated to azide, followed by the IF reaction using anti-HA Ab and anti-mouse IgG-Alexa Fluor 488. Cells were counterstained with DAPI. Arrows and arrowheads indicate cells expressing and not expressing nsp1, respectively. Bar indicates 20 μm. 5-EU fluorescence intensity in nsp1 expressing and non-expressing cells was quantified by Image J. The percentage of the EU signal in nsp1 expressing cells as compared to nsp1 non-expressing cells is shown in the right panel. Ordinary one-way ANOVA with Tukey’s multiple comparisons test. **, p < 0.01. N = 3. (B) Total RNA was extracted from 293T cells co-transfected with FF-Luc cDNA and the indicated nsp1 mRNAs. qRT-PCR was used to measure FF-Luc mRNA and 18S rRNA (as a standard). The quantity of the RNA was normalized to that in the control sample (GFP). Ordinary one-way ANOVA with Dunnett’s multiple comparisons test. ****, p < 0.0001. N = 3. (C) 293T cells were transfected with mRNA for GFP or NL63 nsp1 along with cDNA for FF-Luc under an RNA Pol I (left) or RNA Pol II (right) promoter. Total RNA was extracted, and the absolute copy number of FF-Luc was quantified by qRT-PCR. Unpaired T test. ****, p < 0.0001. ***, p < 0.001. N = 3. (D) 293T cells were infected with SeV Cantel to induce IFN and then transfected with mRNA for the indicated protein. One condition was also treated with ActD to prevent transcription. Eighteen h post transcription, total RNA was extracted, and the absolute copy number of IFN-β mRNA was quantified by qRT-PCR. Ordinary one-way ANOVA with Dunnett’s multiple comparisons test. ****, p < 0.0001. N = 3. All graphs throughout the figure show the mean and standard deviation.

We next determined the effect of nsp1 on host RNA synthesis by a *de novo* RNA synthesis assay measuring 5-ethynyl uridine incorporated into transcripts [23]. 293T cells were transfected with nsp1 mRNA. Six hours later, cells were labeled for 1 h with 5-ethynyl uridine and newly synthesized RNAs were detected by click chemistry. As expected, SARS-CoV-1 and -2 nsp1s did not affect host RNA synthesis (Fig 2A). Similarly, 229E nsp1, which has negligible shutoff activity, did not have visible effects on RNA synthesis. In sharp contrast, cells expressing NL63 nsp1 strongly reduced RNA synthesis at 6 h post transfection. To confirm the effect of NL63 nsp1 on transcription activity, we used qPCR to quantify luciferase mRNA transcribed from transfected cDNA in nsp1-expressing cells. No difference was detected between control cells expressing GFP and cells expressing nsp1 from SARS-CoV-1, SARS-CoV-2, or 229E (Figs 2B and S2). In contrast, we detected a 70-fold decrease in luciferase mRNA in NL63 nsp1-expressing cells compared to GFP-expressing cells.

We further determined whether this inhibition of RNA synthesis by NL63 nsp1 is specific to RNA polymerase II (RNAPII) transcripts. Cells were transfected with NL63 nsp1 or GFP mRNA together with cDNA encoding FF-Luc under either an RNAPI or RNAPII promoter. Quantification of FF-Luc RNA indicated that NL63 nsp1 inhibits transcription by RNAPII, but not RNAPI (Fig 2C). In addition to measuring NL63 nsp1’s effect on reporter gene expression, we tested its effect on the production of the endogenous interferon (IFN) mRNA. IFN is one of the host’s first lines of defense following viral infection, as its induction triggers transcription of hundreds of IFN-stimulated genes that are involved in antiviral defense (reviewed in [24]). We infected 293T cells with Sendai virus Cantel (SeV) to induce IFN-β production and then transfected cells with mRNAs encoding NL63 nsp1 or GFP, or we treated with the transcription inhibitor actinomycin D (ActD). SeV infection induced IFN-β mRNA production, which was significantly reduced by NL63 nsp1. These data strongly suggest that NL63 nsp1 blocks transcription activity of RNAPII or targets transcripts for rapid degradation to induce host shutoff, unlike SARS-CoV nsp1s, which block translation of host mRNA.

### Identification of the domain required for NL63 nsp1 shutoff activity

**Fig 3:**
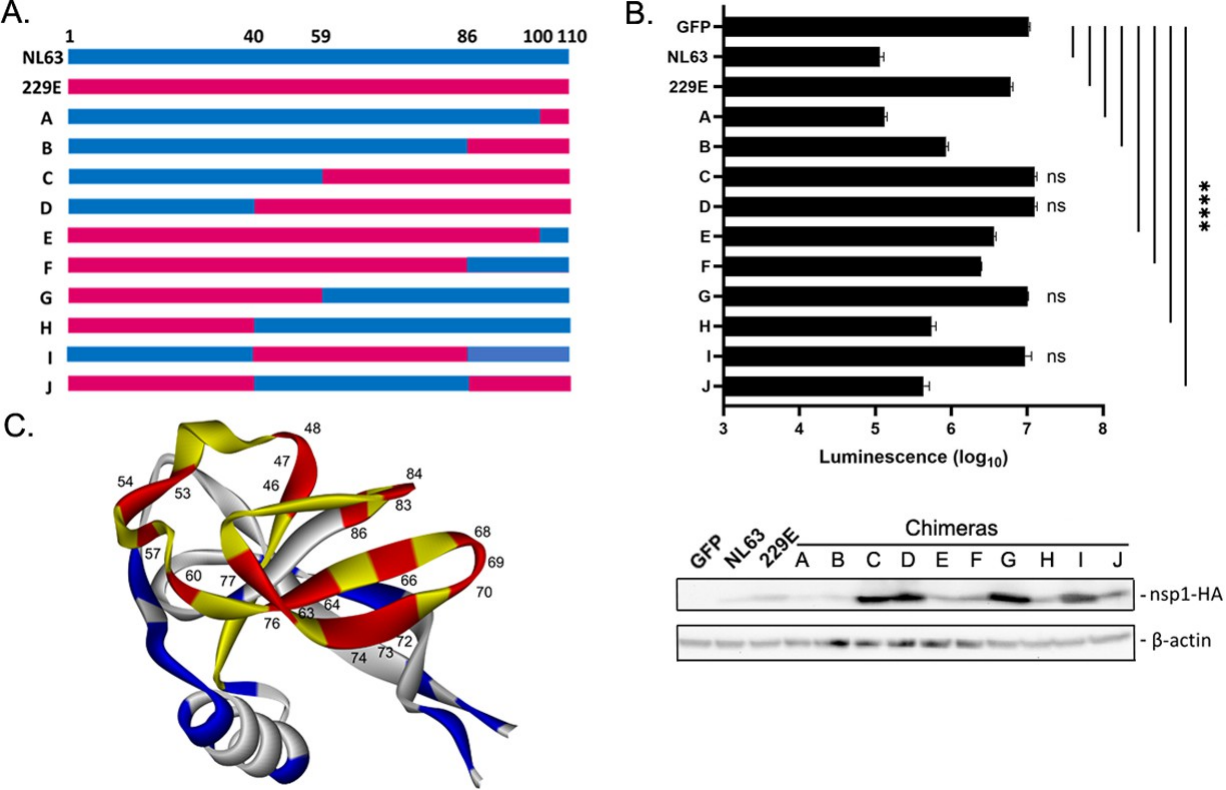
Identification of the domain required for NL63 nsp1 shutoff activity. (A) Schematic diagram showing the nsp1 chimeras. (B) FF luciferase activity of 293T cell lysates transfected with pCAGGS FF-Luc cDNA together with the indicated nsp1 chimeras in pCAGGS. Expression of nsp1 was determined by Western blotting using anti-HA Ab, showing that shutoff activity of nsp1 restricts its own expression. The mean and standard deviation are shown. Ordinary one-way ANOVA with Dunnett’s multiple comparisons test. ****, p<0.0001. N = 3. (C) Predicted 3D structure from Alphafold of NL63 nsp1 highlighting residues 41–86 (yellow), which are required for shutoff activity. Residues different between NL63 and 229E within this key domain are shown in red and those outside the domain are shown in blue.

Although NL63 and 229E nsp1s share relatively low sequence homology (61%), overall protein structures of α-CoV nsp1s are reported to be similar [13]. In cells transfected with nsp1 mRNAs together with FF-Luc DNAs, we detected limited expression of 229E nsp1, suggesting that 229E nsp1 is not as stable as NL63 nsp1 (Fig 1B). Therefore, we next constructed a series of NL63/229E chimeric nsp1 cDNAs and characterized their expression and shutoff activity to identify the functional domain of nsp1 shutoff activity (Figs 3A and S1). We designed the chimeric constructs so as to not interfere with predicted secondary structures of nsp1. We transfected cells with cDNAs for WT or chimeric nsp1 along with FF-Luc and measured the luciferase activity and protein expression (Fig 3B). All the chimeric nsp1s localized in both the nucleus and the cytoplasm, similar to WT NL63 and 229E nsp1s (S3 Fig). In cells transfected with cDNAs, more 229E nsp1 was expressed than NL63 nsp1. However, 229E nsp1’s shutoff activity was much lower than NL63 nsp1, indicating a strong difference in its activity to regulate host protein synthesis. Interestingly, the chimeric proteins containing only the N-terminal half (residues 1–59) or C-terminal half (residues 60–110) from NL63 (chimeras C and G, respectively) showed no shutoff activity, suggesting that the functional domain is in the middle region of the protein. In fact, chimera I, which contained residues 40–86 from 229E nsp1, showed no shutoff activity, while the opposite construct containing residues 40–86 from NL63 nsp1 (chimera J) showed strong shutoff activity. The shutoff activity correlated with the level of nsp1 expressed from transfected cDNAs (Fig 3B, bottom). This is consistent with a previous report of influenza A virus showing that viral shutoff protein PA-X restricts its own expression from cDNA [25]. A structural prediction created in Alphafold [26] revealed that residues 40–86 are within the strands β3, β4, β5, β6, and β7 [11], and residues different between NL63 and 229E in this region are exposed on the surface of the protein. These results suggest that this specific domain plays a role in inducing shutoff through interaction with one or more host protein(s) (Fig 3C).

### Degradation of RNAPII in NL63 nsp1 expressing cells

**Fig 4:**
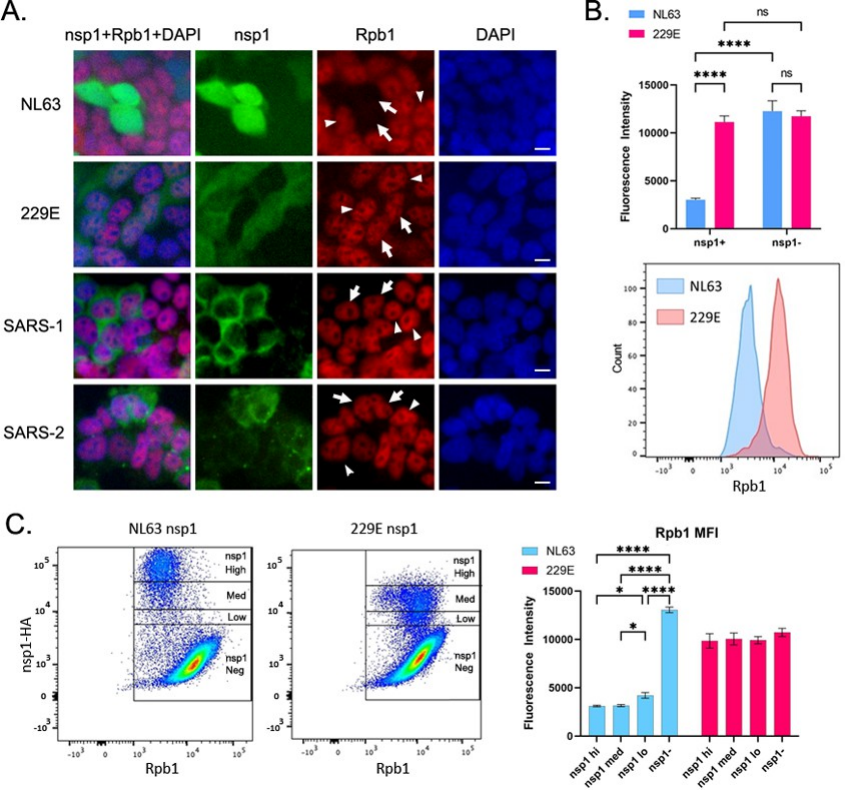
NL63 nsp1 induces degradation of RNAPII. (A) 293T cells were transfected with mRNA for the indicated nsp1-HA. At 18h post transfection, cells were fixed and stained for nsp1-HA and Rpb1 using anti-HA and anti-Rpb1 NTD Abs and analyzed by IF. Images were captured using 40x objective. Arrows and arrowheads indicate nsp1 expressing and non-expressing cells, respectively. Bar indicates 20 μm. (B) 293T cells transfected as in (A) were analyzed by flow cytometry. The graph on top represents the mean and standard deviation of the MFI of Rpb1 NTD in cells expressing or not expressing nsp1. A representative histogram of cells expressing nsp1 is shown below. Significance was tested with an ordinary one-way ANOVA with Ŝidák’s multiple comparisons test. ****, p < 0.0001. N = 3. (C) Representative flow plots of 293T cells analyzed by flow cytometry as in (B). The fluorescence intensity of Rpb1 NTD is shown on the x-axis, and the fluorescence intensity of nsp1 is shown on the y-axis. The graph shows the mean and standard deviation of the MFI of Rpb1 in nsp1-high, -medium and -low populations. Significance was tested with a two-way ANOVA with Tukey’s multiple comparisons test. *, p < 0.05. ****, p < 0.0001. N = 3.

Because NL63 nsp1 specifically inhibited gene expression under the RNAPII promoter (Fig 2C), we next determined the impact of nsp1 on RNAPII expression. We transfected 293T cells with *in vitro* synthesized mRNA for NL63, 229E, SARS-CoV-1 or SARS-CoV-2 nsp1 and then detected the catalytic subunit of RNAPII, Rpb1, by IF assay using an antibody (Ab) against the N-terminal domain (NTD) of Rpb1. While 229E, SARS-CoV-1, and SARS-CoV-2 nsp1s had no visible effect on Rpb1 levels, NL63 nsp1 dramatically reduced Rpb1 levels (Fig 4A). Because none of the other nsp1s tested had an effect on Rpb1 expression, we used only 229E nsp1 for comparison going forward. To quantify the effects of nsp1 on Rpb1, we analyzed Rpb1 expression in nsp1-expressing cells by flow cytometry (Fig 4B). Cells expressing NL63 nsp1 had a 4.1-fold decrease in Rpb1 median fluorescence intensity (MFI) compared to cells not expressing nsp1, while there was no change in Rpb1 MFI in cells expressing 229E nsp1 compared to cells not expressing nsp1. Interestingly, within transfected cells, nsp1 fluorescence intensity did not correlate to Rpb1 fluorescence intensity (Fig 4C). Even cells expressing low levels of NL63 nsp1 effectively reduced Rpb1 expression. These results suggest that NL63 nsp1 triggers a cellular pathway to degrade Rpb1 instead of directly degrading it.

### NL63 nsp1 degrades Rpb1 in a proteasome-dependent manner

**Fig 5:**
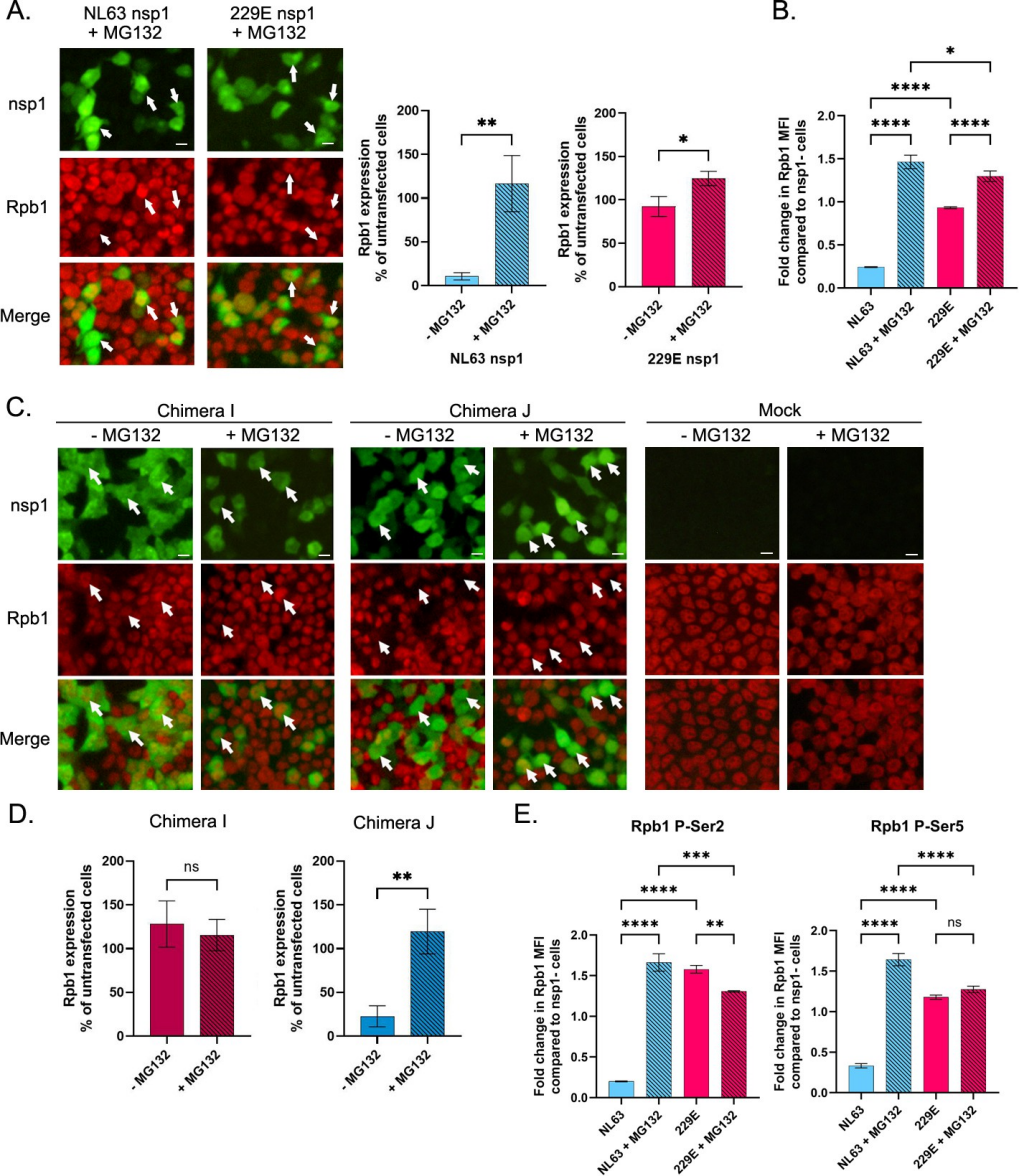
NL63 nsp1 degrades Rpb1 in a proteasome-dependent manner. (A) 293T cells were transfected with mRNA for the indicated nsp1-HA, and MG132 was added at the time of transfection. Eighteen h post transfection, cells were fixed and stained for nsp1-HA and Rpb1 NTD. Cells were then analyzed by IF. Images were captured using 20x objective. Arrows in IF images indicate cells expressing nsp1. Bar indicates 20 μm. The signals for Rpb1 here and from Fig 4 were quantified by Image J and compared with those of non-expressing cells. N = 3. (B) 293T cells were transfected as in (A) and analyzed by flow cytometry. The graph represents the fold change in Rpb1 NTD MFI in nsp1+ cells to that of nsp1- cells from the same condition. N = 3. (C and D) 293T cells were analyzed by IF as in Figs 4A and 5A, except chimeric nsp1s were used. The signals for Rpb1 were quantified by Image J and compared with those of non-expressing cells in D. N = 3. (E) 293T cells were analyzed by flow cytometry as in Fig 5B, except cells were stained for Rpb1 Phospho-Ser2 (left) or Rpb1 Phospho-Ser5 (right). N = 3. All bar graphs represent the mean and standard deviation. Significance was tested with an ordinary one-way ANOVA with Ŝidák’s multiple comparisons test (B, E) or unpaired t test (A, D). ****, p < 0.0001. ***, p < 0.001. **, p < 0.01. *, p < 0.05. ns, not significant.

It is well recognized that transcriptional stress or DNA damage can cause elongating RNAPII to stall during transcription. If cells are unable to relieve stalled RNAPII, the “last resort” pathway turns on, and stalled Rpb1 is ubiquitinated and subsequently degraded by the 26S proteasome [27]. To determine whether NL63 nsp1 induces a similar response and causes degradation of Rpb1 in a proteasome-dependent manner, we transfected cells with NL63 nsp1 mRNA and cultured them in the presence of proteasome inhibitor MG132. Rpb1 levels were then measured by IF and flow cytometry analysis. Both assays showed that addition of MG132 rescued Rpb1 expression (Fig 5A and 5B). We further confirmed this using chimeric nsp1 proteins I and J, which contain the key domain from 229E and NL63, respectively (Fig 3A). Consistent with the results of shutoff activity, chimera J reduced Rpb1 expression in transfected cells while chimera I did not (Fig 5C and 5D). This loss of Rpb1 by chimera J was suppressed by MG132, which was also observed for NL63 nsp1. These results suggest that NL63 nsp1 induces proteasome-dependent RNAPII degradation.

Within RNAPII, the CTD of Rpb1 coordinates transcription and RNA processing [28]. The CTD consists of 52 heptapeptide repeats (YSPTSPS) which can be phosphorylated. The dynamic phosphorylation status of these residues is responsible for the specific recruitment of numerous regulatory proteins. Ser5 phosphorylation is linked to recruiting both elongation factors and RNA processing proteins. As elongation proceeds, Ser5 phosphorylation levels fall, while Ser2 phosphorylation levels rise [29]. To determine if Rpb1 degradation by nsp1 is specific to Rpb1 phosphorylation status, we repeated the above assays using Abs specific to the Rpb1 CTD phosphorylated at either Ser2 or Ser5. Reduction of the specific states of Rpb1 was quantified by flow cytometry. Compared to cells not expressing nsp1, NL63 nsp1 reduced Rpb1 levels by 5.0- or 3.0-fold for phospho-Ser2 or phospho-Ser5, respectively (Fig 5E). These values are similar to the level of reduction detected when using the anti-Rpb1 NTD Ab (4.1-fold) (Fig 5B), which suggests that nsp1-induced Rpb1 degradation is not dependent on its transcriptional status.

### Rpb1 is ubiquitinated and degraded in NL63-infected cells

**Fig 6:**
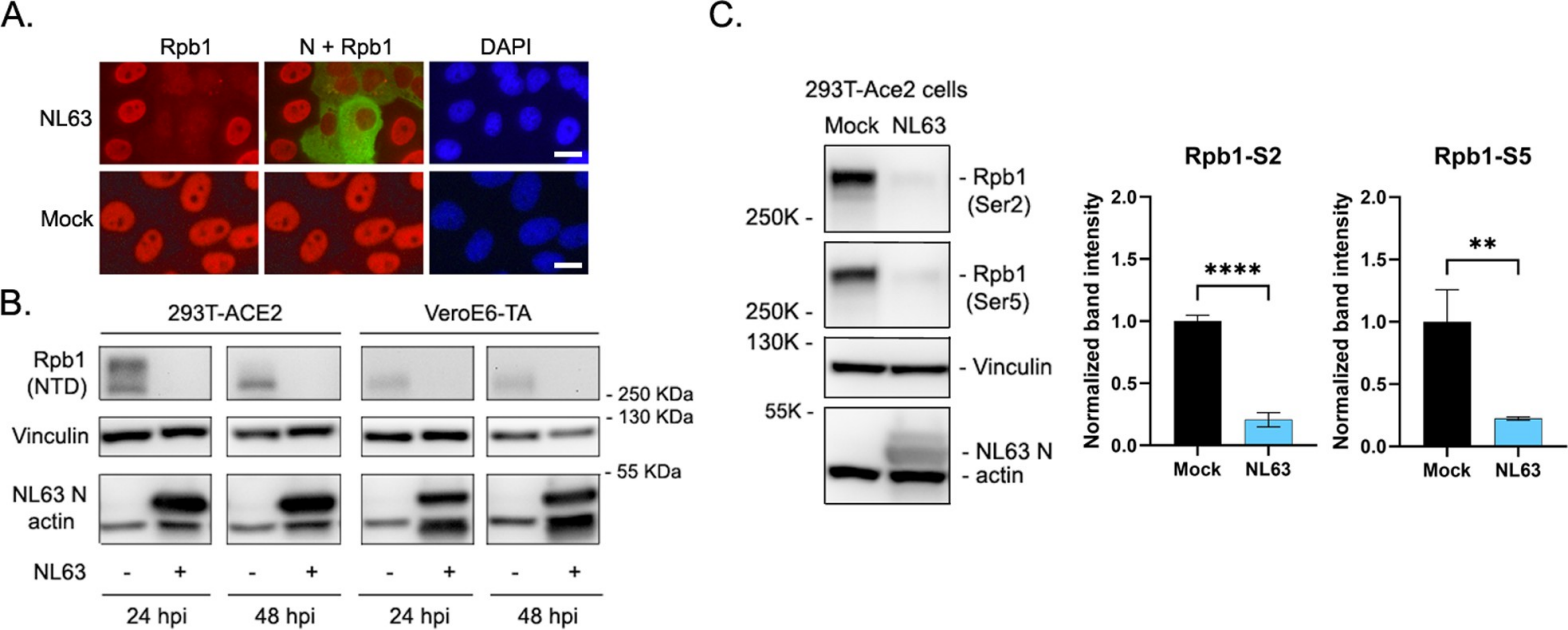
Rpb1 is degraded in NL63-infected cells. (A) VeroE6-TA cells infected with NL63 at an MOI of 0.5 were fixed and stained for NL63 N and Rpb1 CTD (non-phospho-specific) 24 hpi. Images of mock infected or NL63 infected cells were captured using the 40x objective. Bar indicates 20 μm. (B) 293T-ACE2 and VeroE6-TA cells were infected with NL63 at an MOI of 0.5. At the indicated time post-infection, cells were lysed and analyzed for the expression of Rpb1 by Western blot using anti-Rpb1-NTD Ab. Vinculin and actin were included as loading controls. (C) 293T-ACE2 cells were infected with NL63 at an MOI of 0.5. Twenty-four hpi, cells were lysed and analyzed for Rpb1 expression by Western blot using the indicated Ab. Band intensity was calculated by Image J. Unpaired t test. ****, p < 0.0001. **, p < 0.01. N = 3.

To confirm Rpb1 degradation in the context of NL63 infection, we used Vero E6-TMPRSS2-T2A-ACE2 (VeroE6-TA), 293T-ACE2, and LLC-MK2-ACE2 cells, all of which overexpress human ACE2 (the receptor for NL63) and are susceptible to viral infection. First, we infected VeroE6-TA cells with NL63 and analyzed expression of Rpb1 by IF assay. NL63 infection clearly reduced the expression of Rpb1 (Fig 6A). Degradation of Rpb1 was confirmed by Western blot analysis using Ab against the NTD of Rpb1 (Fig 6B). We also analyzed infected cell lysate via Western blot with additional Abs which detect Rpb1 CTD (both phosphorylated and unphosphorylated forms of the protein), Rpb1 CTD phospho-Ser2, or Rpb1 CTD phospho-Ser5. Similar to what we observed in nsp1-transfected cells, NL63 infection induced degradation of Rpb1 regardless of transcriptional status (Fig 6C).

**Fig 7:**
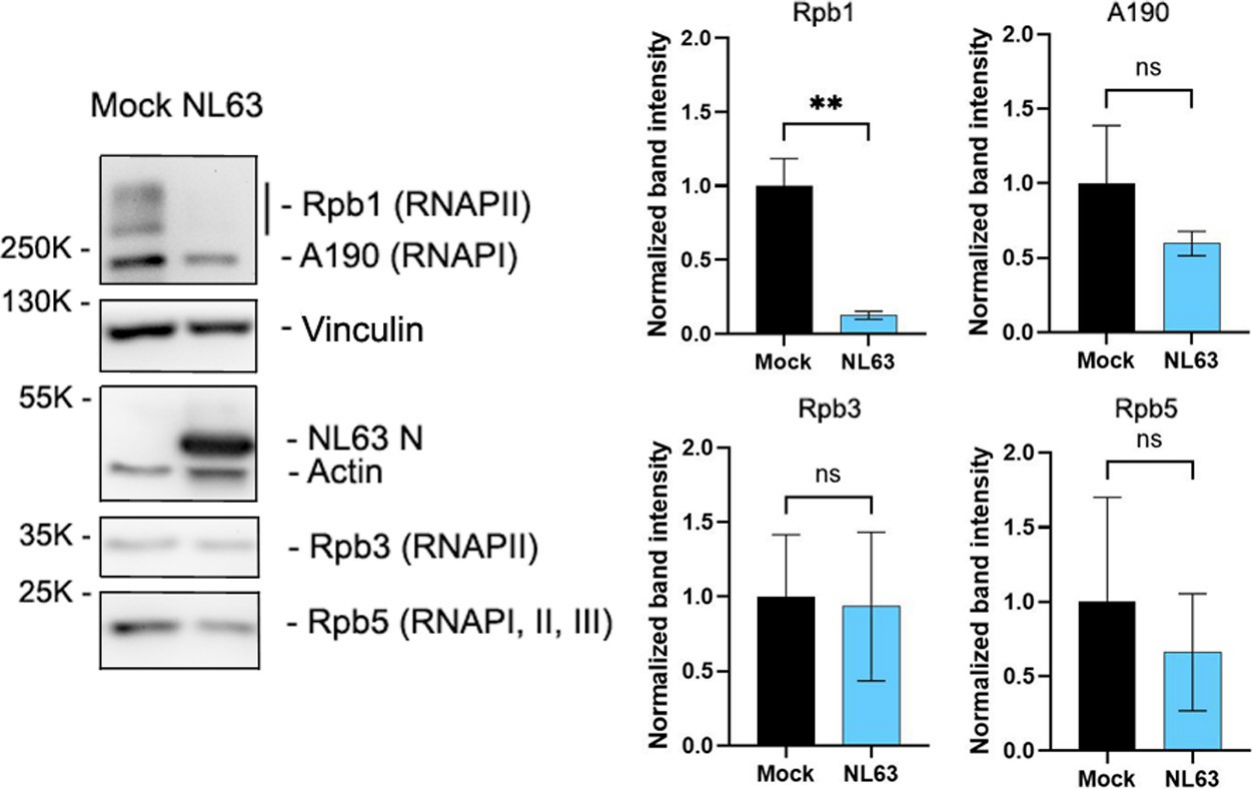
Specific degradation of Rpb1 in NL63-infected cells. (A) 293T-ACE2 cells were infected with NL63 at an MOI of 0.5. At 24 hpi, cells were lysed and analyzed for the expression of RNAP components by Western blot using Abs specific for Rpb1-NTD, A190, Rpb3, or Rpb5. Vinculin and actin were included as loading controls. Band intensity was determined by Image J and normalized to vinculin. Unpaired t test. **, p < 0.01. ns, not significant. N = 3.

Next, we determined the effect of NL63 infection on the stability of other RNA polymerase components. 293T-ACE2 cells infected with NL63 were analyzed for the expression of RNAPII components Rpb1 and Rpb3, RNAPI subunit A190, and Rpb5, which is a component of all three RNAPs. The results showed that although NL63 infection significantly reduces the level of Rpb1, the expression of Rpb3 was not affected, suggesting that nsp1 specifically targets Rpb1 to block transcription by RNAPII (Fig 7). NL63 infection also did not significantly affect the expression of the catalytic component of RNAPI, A190, which is consistent with our results of luciferase gene expression under the RNAPI promoter (Fig 2C). Although it was not significant, we detected a slight reduction of both A190 and Rpb5. Because RNAPII is responsible for production of the polymerase components, it is likely that the degradation of Rpb1 and subsequent lack of production of RNAPII complexes negatively impacts the production of other polymerase proteins, although the level of existing proteins is not affected. These results suggest that NL63 nsp1 specifically targets Rpb1 for degradation.

**Fig 8:**
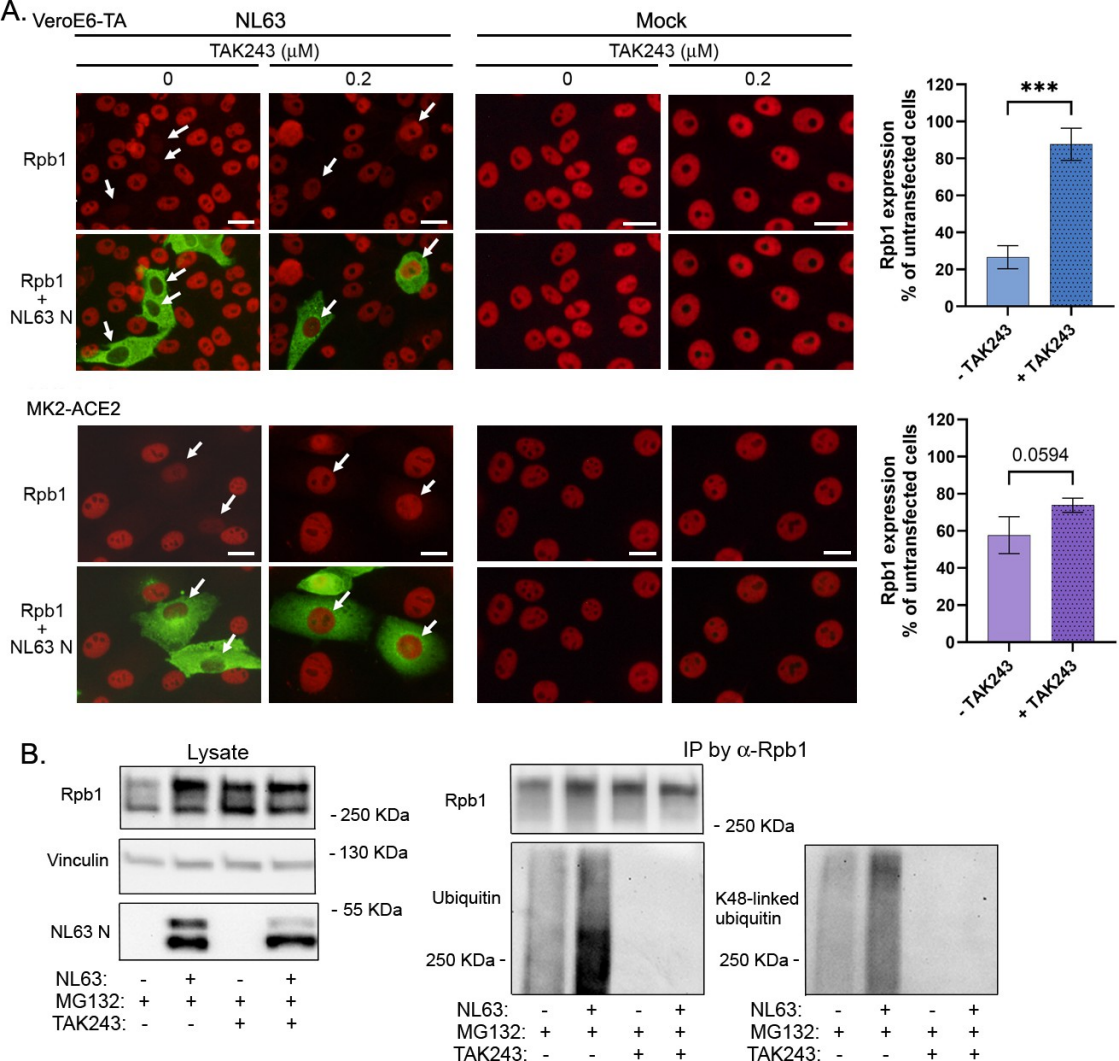
Ubiquitination is required for Rpb1 degradation in NL63-infected cells. (A) VeroE6-TA and LLC-MK2-ACE2 cells were infected with NL63 or mock and treated with 0.2 μM TAK243. At 24 hpi, cells were fixed and processed for IF with the indicated Abs. Images were captured using the 40x objective. Arrows indicate infected cells. Bar indicates 20 μm. The signals for Rpb1 were quantified by Image J and compared with those of non-expressing cells. Unpaired t test. ***, p < 0.001. N = 3. (B) 293T-ACE2 cells infected as in Fig 6 were treated with 10 μM MG132 and 0.2 μM TAK243 as indicated. At 24 hpi, cells were lysed and incubated with anti-Rpb1 CTD Ab for immunoprecipitation. Lysates and immunoprecipitated materials were analyzed by Western blot with the indicated Ab.

To determine the involvement of ubiquitination in the degradation of Rpb1, we treated infected Vero E6-TA and MK2-ACE2 cells with TAK243, an inhibitor of ubiquitin activating enzyme, the primary mammalian E1 enzyme that regulates the ubiquitin conjugation cascade [30,31]. IF analysis showed that TAK243 rescued Rpb1 expression in infected cells (Fig 8A). Loss of Rpb1 in NL63 infected cells was less clear in MK2-ACE2 cells, although TAK243 treatment did increase the level of Rpb1 in NL63-infected cells. To confirm this result, we treated infected cells with MG132 and performed immunoprecipitation of Rpb1. The ubiquitination of Rpb1 was analyzed by Western blot analysis using Ab for either total or K48-linked ubiquitin, which is the canonical signal for proteasomal degradation [30]. The results indicate enhanced ubiquitination, both total and K48-linked, of Rpb1 in NL63 infected cells (Fig 8B). This ubiquitination was not detected in cells further treated with TAK243. The lower band of N detected in this experiment could be a proteolytically cleaved product, as reported for PEDV [32]. Overall, these data indicate that human CoV NL63 nsp1 triggers ubiquitination and proteasomal degradation of Rpb1 to shut off host protein synthesis.

## Discussion

Many viruses evade host innate and acquired immune responses by blocking host protein expression. Host shutoff is an effective strategy to block host innate responses [33]. In this study, we have uncovered a novel mechanism of host shutoff by human CoV NL63 nsp1, which targets and degrades host RNAPII by inducing its ubiquitination and proteasomal degradation. Our data indicate that the same protein from viruses within the same family uses completely different approaches to regulate host antiviral responses. Nsp1 is best characterized from SARS-CoV-1 and -2 and was shown to have multiple functions to control host protein expression. Structural and biochemical analysis revealed that SARS-CoV nsp1s block the mRNA entry tunnel of 40S ribosomal subunits as their main mechanism of host shutoff [17,18]. SARS-CoV-2 nsp1 is also reported to interact with the mRNA export receptor NXF1-NXT1 and prevent proper nuclear export of host mRNAs [22,34]. In addition, SARS-CoV-2 nsp1 promotes host mRNA degradation when bound to host ribosomes [19,20,34,35]. It initiates cleavage near the 5’ end of mRNAs in a sequence-dependent manner through a possible recruitment of a host endonuclease that results in subsequent digestion of mRNA by the host exonuclease Xrn1 [19–21]. Consistent with its localization, the nsp1s from SARS-CoVs function mainly in the cytoplasm to induce host shutoff. A recent study further supports the importance of ribosome binding in nsp1-mediated shutoff by β-CoVs, including human CoVs OC43 and HKU1 [36]. Similar to SARS-CoVs, MERS-CoV nsp1 also induces shutoff by blocking host mRNA translation and degradation of mRNAs [37]. However, MERS-CoV nsp1 localizes in both the nucleus and the cytoplasm, and it has been reported to induce host mRNA degradation without binding to the ribosome. This suggests that MERS-CoV nsp1 uses different strategies from SARS-CoV nsp1s to degrade host mRNAs and block translation.

Interestingly, the key residues for translational inhibition and mRNA degradation of these β-CoV are located within the CTD, which does not exist in α-CoV nsp1s. It has been previously shown that α-CoV nsp1s do not bind host ribosomes [36], and we confirmed NL63 nsp1’s lack of ribosome association (Fig 1C). So far, limited studies have been done on the mechanism of human α-CoV nsp1-mediated shutoff and its role in infection. However, studies on non-human α-CoVs, such as transmissible gastroenteritis virus (TGEV), porcine epidemic diarrhea virus (PEDV), swine acute diarrhea syndrome coronavirus (SADS-CoV), and feline infectious peritonitis virus (FIPV) showed that these nsp1 proteins share an overall structural similarity despite minimal sequence conservation [12–14]. TGEV nsp1 efficiently suppressed protein synthesis in mammalian cells, although it was unable to bind 40S ribosomal subunits or promote host mRNA degradation. TGEV nsp1 was also able to suppress protein translation in cell-free HeLa cell extract, but not in rabbit reticulocyte lysate, suggesting that TGEV nsp1 inhibits translation through its specific interaction with mammalian host factor(s) [38]. It was also reported that TGEV nsp1 required amino acids 91–95 for its shutoff function, which is outside of the region we identified for NL63 nsp1 (Fig 3). Among the α-CoV, 229E and PEDV are the most closely related to NL63 and together form α-CoV group 1b [3]. PEDV nsp1 was reported to promote the proteasomal degradation of CBP and NF-κB to inhibit the interferon response. The detailed mechanism of PEDV nsp1 in inhibiting host protein synthesis is unclear [39,40]. Also, it is not known whether it targets RNAPII for degradation. However, the functional domain of PEDV nsp1 involved in host shutoff is located close to the domain we identified in NL63 nsp1 [41].

In this study, we found that NL63 nsp1 triggers ubiquitination and proteasomal degradation of Rpb1. The mechanism by which NL63 nsp1 induces ubiquitination and degradation of Rpb1 is currently unknown. It is possible that NL63 nsp1 is directly involved in the specific interaction of an E3 ubiquitin ligase with Rpb1. We immunoprecipitated NL63 nsp1 in transfected cells to determine the possible complex formation, but we failed to detect Rpb1 in the pulldown materials (S4 Fig). Because of the limit of detection, we cannot exclude the possibility of an interaction between nsp1 and Rpb1. However, we found that the quantity of nsp1 within the cells does not correlate with the level of Rpb1 degradation (Fig 4C), which may suggest that nsp1 activates cellular pathway(s) to induce ubiquitination of the catalytic component of RNAPII. Although this has not been previously recognized for CoVs, host shutoff by targeting RNAPII has been reported for arthropod-borne Old World alphaviruses and bunyaviruses, although the precise mechanisms of Rpb1 degradation remain unclear [42–44]. Degradation of Rpb1 was shown to be induced by nsP2 proteins from Sindbis virus (SINV), Semliki Forest virus (SFV), and chikungunya virus (CHIKV), and this seems to be conserved across Old World alphavirus species [42]. Unlike the classically understood “last resort” pathway used by cells to clear stalled RNAPII, Old World alphaviruses target both non-phosphorylated and phosphorylated Rpb1 for degradation, similar to what we observed for NL63 nsp1 [42]. However, NSs of La Crosse virus, a member of the Bunyaviridae family, specifically targets and degrades elongating hyperphosphorylated Rpb1, suggesting the presence of various approaches to degrade RNAPII by viral proteins [44].

Our findings imply that structurally similar small nonstructural proteins from different CoVs regulate host antiviral responses using distinct mechanisms. The effectiveness of targeting transcription or translation needs further analysis to understand why different viruses take distinct strategies to control host responses. It is also interesting that nsp1 from the other human α-CoV 229E showed weak shutoff activity. We noticed that 229E nsp1 is less stable than NL63 nsp1, especially when expressed from transfected mRNAs. This may indicate that it requires interaction with other viral proteins for stable expression. Even though both NL63 and 229E cause mild respiratory disease in humans, their approach to regulate host antiviral responses could be different. 229E may take an alternative approach to regulate host innate responses by nsp1 or other viral protein(s), such as nsp14 [45]. All human CoVs originated from bats or rodents, and most of them spilled over to humans via different intermediate hosts [46]. It is possible that the viruses developed alternative approaches during evolution within different hosts to better control antiviral responses and achieve efficient virus transmission and maintenance in specific hosts. Further analysis on the role of nsp1 in virus growth within various species may highlight the impact of host shutoff strategies on host adaptation.

## Materials and methods

### Cell lines and viruses

293T (ATCC: CRL-3216), A549 (ATCC, CCL-185), Vero E6-TMPRSS2-T2A-ACE2 (VeroE6-TA, BEI resource: NR-54970), and LLC-MK2 (ATCC, CCL-7) cells were maintained in Dulbecco’s modified Eagle’s medium (DMEM, Gibco) supplemented with 8% fetal bovine serum (FBS, Avantor), 25 mM HEPES (Gibco), and 50 μg/ml gentamicin (Gibco). 293T-ACE2 cells, which were transformed with pCEP4-myc-ACE2 (Addgene: 141185), were produced and provided by Dr. Serra-Moreno (University of Rochester). LLC-MK2 cells overexpressing human ACE2 were generated by transfecting LLC-MK2 cells with the pCEP4-myc-ACE2 plasmid and selecting with 400 μg/mL of hygromycin B (Invitrogen). NL63 virus was obtained from BEI resources (FR-304) and propagated in LLC-MK2-ACE2 cells in DMEM supplemented with 2% FBS, 25 mM HEPES, and 50 μg/mL gentamicin. Sendai virus Cantel strain was propagated in 10-day-old embryonated chicken eggs.

### Plasmids and in vitro transcribed RNA

Nsp1 genes with a C-terminal HA tag and the 5’UTR from the CoV were cloned into a pCAGGS expression vector containing a T7 promoter. Nsp1 genes from 229E and NL63 were cloned from viruses obtained from BEI resources (229E: FR-303, NL63: FR-304). SARS-CoV-2 RNA obtained from BEI resources (NR-52285) was used to clone its nsp1, and SARS-CoV nsp1 cDNA was obtained directly from BEI resources (NR-15182). Chimeric 229E/NL63 nsp1s in pCAGGS vector were created via overlapping PCR followed by subcloning. The pPolI-NP-Luc plasmid, which contains the FF Luc gene under the control of the human RNA polymerase I promoter, was obtained from T. Wolff (Robert-Koch Institute, Berlin, Germany). The pCAGGS-Luc plasmid was constructed by subcloning the FF Luc gene from pPolI-NP-Luc to the pCAGGS vector. pRL-SV40 was obtained from Promega. pTF1-eGFP was produced by subcloning the eGFP gene from pEGFP-N1 (Clontech) to pTF1 plasmid which contains T7 promoter [47]. Capped and polyadenylated RNAs were synthesized *in vitro* using the HiScribe T7 ARCA mRNA Kit (with tailing) (New England BioLabs: E2060S) according to the manufacturer’s protocol.

### Polysome fractionation

293T cells in 100 mm dishes were transfected with 16 μg of cDNA for nsp1 as indicated using Lipofectamine 2000 in Opti-MEM for 24 h at 37°C. Polysome fractionation was performed as described previously [48]. Briefly, 24 h post transfection, cycloheximide (CHX, Thermo Fisher) was added to cells at a final concentration of 100 μg/mL for 10 minutes. Cells were washed three times with ice cold PBS containing 100 μg/mL CHX. Cells were then lysed in 750 μL of high salt lysis buffer (300 mM NaCl, 20 mM Tris-HCl pH 7.5, 10 mM MgCl2, 1x HALT protease inhibitor, 120 units rRNAsin, and 100 μg/mL CHX) on ice for 15 minutes with occasional mixing and vortexing, and lysates were centrifuged at 12,000 x g for 10 min. Supernatants were transferred to fresh tubes, and total RNA concentration was determined by a NanoDrop 2000. A total of 300 μg of RNA was loaded on top of a 10–50% linear sucrose gradient prepared in high salt lysis buffer and centrifuged for 90 minutes at 39,000 rpm at 4°C using a Beckman Coulter SW 41Ti rotor. Gradients were fractionated using a BR-188 Density Gradient Fractionation System (Brandel) with absorbance measured at 254 nm. Following fractionation, proteins were precipitated using deoxycholate (DOC) and trichloroacetic acid (TCA) according to the protocol by Koontz [49]. Precipitated proteins were analyzed by western blot.

### Luciferase assay

Luciferase assays were conducted in 293T cells in 12-well plates. Cells were transfected with 0.4 μg of pCAGGS-Luc and 0.4 μg of cDNA or mRNA of the indicated gene using Lipofectamine 2000 (Invitrogen) in Opti-MEM (Gibco) for 16 h (with RNA) or 24 h (with DNA) at 37°C. Luciferase activity was measured by the dual-luciferase reporter assay system (Promega).

### Immunofluorescence

Transfected or infected cells were fixed with 4% paraformaldehyde (PFA) in PBS and permeabilized with 0.5% Triton-X in PBS. Cells were then incubated with primary Ab in 0.1% BSA/PBS for 1 h at RT. Primary Abs used were mouse anti-HA (Invitrogen: 26183), rabbit anti-Rpb1 NTD D8L4Y (Cell Signaling Technology: 14958), mouse anti-Rpb1 CTD (BioLegend: 904001), or rabbit anti-NL63 N (Sino Biological: 40641-T62). Following primary Ab reaction, cells were washed and incubated with secondary Ab and DAPI for 1 h at RT. Secondary Abs were anti-mouse IgG-Alexa Fluor 488 (Invitrogen: A11029) and anti-rabbit IgG Alexa Fluor 594 (Jackson ImmunoResearch Laboratories: 111-585-144). Fluorescent images were acquired using an Olympus IX 50 inverted fluorescence microscope with an objective lens as described in figure legends.

### 5-EU Labeling and click chemistry

293T cells were transfected with nsp1 mRNA as above and newly synthesized RNA was detected using Click-iT RNA imaging kit (ThermoFisher: C10330) according to the kit directions. Briefly, 6 h after transfection, 1 mM 5-ethynyl-uridine was added to culture media for 1 h. Cells were then fixed and processed for IF as above.

### qRT-PCR

For measuring total mRNA, cell lysates were treated with TRIzol Reagent (ThermoFisher: 15596026), and RNA was extracted according to the manufacturer’s protocol. The RNA concentration and purity were determined with a Nanodrop 2000 (ThermoFisher). For RT-PCR reaction, 1 μg of total RNA was digested with DNase I (New England BioLabs) and then purified with TRIzol reagent. One hundred ng of purified RNA was used for RT-PCR using RevertAid Reverse Transcription Kit (ThermoFisher: K1691). Real-time PCR was carried out using SYBR Green PCR Master Mix (ThermoFisher) with an Applied Biosystems 7300 Real-time PCR system. DNA standards for FF-luciferase mRNA, PolI luciferase mRNA, IFN-β mRNA, and 18S rRNA (as a control) were generated by PCR. A standard curve from 10^1^ to 10^9^ copies was used for quantification. To detect IFN-β mRNA, 293T cells were infected with SeV at an MOI of 5 for 1 h. The viral inoculum was removed and replaced with the transfection inoculum ± 5 μM Actinomycin D (Sigma), and cells were treated with TRIzol Reagent 18 h post transfection. qRT-PCR was completed as described above.

### Western blots and immunoprecipitation

For both Western blot and immunoprecipitation, cells were lysed in RIPA lysis buffer containing Halt protease inhibitor and phosphatase inhibitor (ThermoFisher Scientific: PI87785 and PI78420), and 50 μM MG132 (MedChemExpress: HY-13259). For the detection of Rpb1, lysates were sonicated using a Branson probe sonicator for 10 seconds at 20% output. For immunoprecipitation, 293T-ACE2 cells in 6 well plates were infected with NL63 for 4 h at 34°C. Cells were then treated with 10 μM MG132 with or without 0.2 μM TAK-243 (MedChemExpress: HY-100487) overnight. After lysis, cell lysate was incubated overnight at 4°C with mouse anti-Rpb1 CTD (BioLegend: 904001, 1 μg/sample) conjugated to Protein G Dynabeads (ThermoFisher Scientific: 10003D). IP material was eluted with 2X NuPAGE LDS sample buffer and 5% β-mercaptoethanol. For immunoblotting, whole cell lysates were mixed with 4X NuPAGE LDS sample buffer (ThermoFisher Scientific: NP0007) and 5% β-mercaptoethanol. Lysates were resolved by SDS-PAGE, transferred to 0.45 μM PVDF membranes, and reacted with Abs. Primary Abs used were the same as for IF, with the addition of mouse anti-β-actin (Cell Signaling Technology: 3700), rabbit anti-vinculin (Cell Signaling Technology: 13901), rabbit anti-ubiquitin (Cell Signaling Technology: 43124), rabbit anti-K48-linked ubiquitin (Millipore Sigma: ZRB2150), rabbit anti-phospho-Rpb1 (Ser2) (Cell Signaling Technology: 13499), rabbit anti-phospho-Rpb1 (Ser5) (Cell Signaling Technology: 13523), and mouse anti-Rpb1 CTD. Secondary Abs used were rabbit anti-mouse IgG HRP-linked (Cell Signaling Technology: 7076) and goat anti-rabbit IgG HRP-linked (Cell Signaling Technology: 7074).

### Flow cytometry

Cells were transfected with nsp1 mRNA and treated with 60 μM MG132. After 16 h, cells were trypsinized, stained with Live/Dead Aqua (Invitrogen: L34965) for viability, and fixed with 4% paraformaldehyde (PFA) in PBS for 15 min at RT. Cells were then permeabilized and stained with primary Ab using with 0.5% Triton-X in PBS at RT for 30 min. Primary Abs used rabbit were mouse anti-HA, rabbit anti-Rpb1 NTD D8L4Y, rabbit anti-phospho-Rpb1 (Ser2), or rabbit anti-phospho-Rpb1 (Ser5). Following reaction with primary Ab, cells were incubated with secondary Ab for 30 min at RT. Secondary Abs used were the same as for IF. Flow cytometry analysis was performed on a BD LSR Fortessa, and data was analyzed using FlowJo.

### Image quantification

For both IF and western blot images, pixel density was measured using ImageJ software. For western blots, band intensity for three independent replicates was normalized to that of the loading control for the same sample, and then infected samples were normalized to the mock condition. For IF images, pixel intensity was measured in 20 infected or transfected cells and 20 uninfected or untransfected cells in each of three independent replicates. Data is represented as the average pixel intensity in infected or transfected cells compared to the intensity in uninfected or untransfected cells.

## Supporting information

S1 Fig Amino acids of nsp1 proteins used in the study. Top, the amino acid sequences of nsp1 from each CoV used in the study (NL63, 229E, SARS-CoV-1, and SARS-CoV-2). Bottom, residues from NL63 and 229E nsp1 in the chimeric constructs shown in Fig 3.
(TIF)

S2 Fig Quantified RNA from nsp1-transfected cells. The absolute copy number of FF-Luc mRNA (left) and 18S rRNA (right) from the experiment performed in Fig 2B. Ordinary one-way ANOVA with Dunnett’s multiple comparisons test. ****, p < 0.0001. N=3. For 18S rRNA, no comparisons were statistically significant.
(TIF)

S3 Fig Localization of chimeric nsp1s. cDNA for chimeric nsp1s described in Fig 3 were transfected into 293T cells and visualized by IF as in Fig 1D. Cell nuclei were counterstained with Hoechst 33342. Images were captured using the 40x objective.
(TIF)

S4 Fig Pull down assay of nsp1 with Rpb1. 293T cells pretreated with 5 μM MG132 for 3 hrs were transfected with mRNAs for NL63 or 229E nsp1 or GFP (control). Twenty h after transfection, cell lysates were prepared and applied for immunoprecipitation with anti-HA Ab and analyzed by Western blotting with the indicated Ab.
(TIF)

S1 Data Datasheet containing raw data of this manuscript. (XLSX)
