# Supplementary figures and images for: Human coronavirus NL63 nsp1 induces degradation of RNA polymerase II to inhibit host protein synthesis
Source: PLoS Pathog. 2024 Jun 20;20(6):e1012329. doi: 10.1371/journal.ppat.1012329 (PMC11218958; doi:10.1371/journal.ppat.1012329)

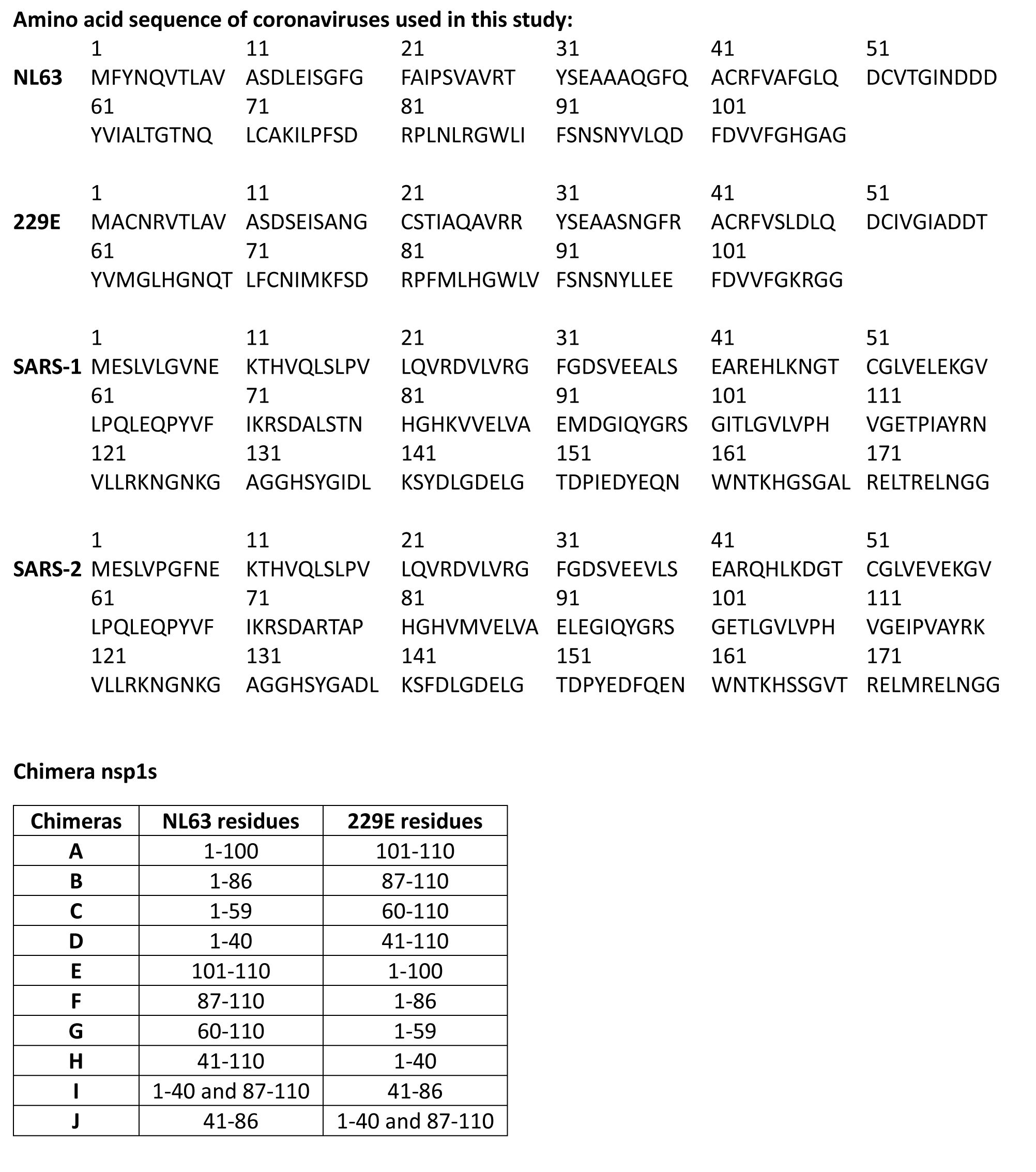

Supplement: S1 Fig — Top, the amino acid sequences of nsp1 from each CoV used in the study (NL63, 229E, SARS-CoV-1, and SARS-CoV-2). Bottom, residues from NL63 and 229E nsp1 in the chimeric constructs shown in Fig 3. (TIF) [file ppat.1012329.s001.tif]

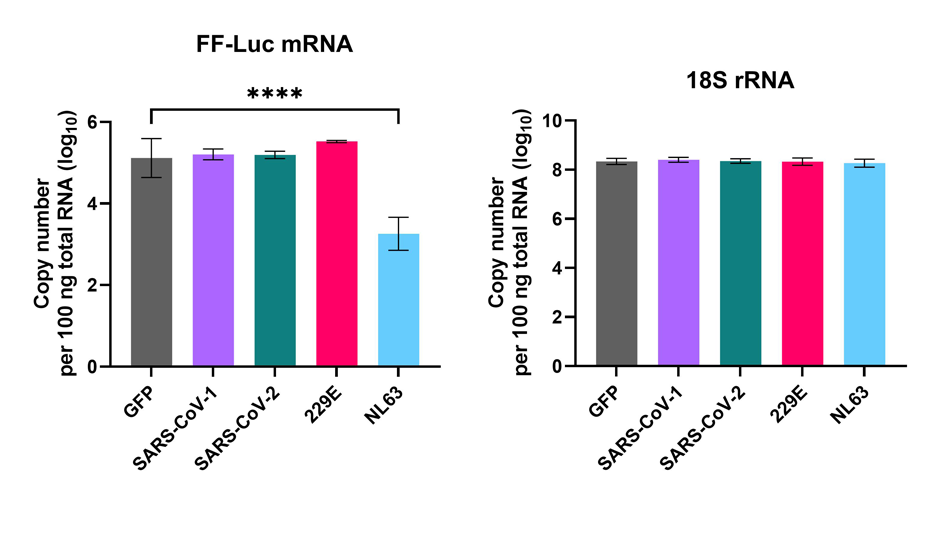

Supplement: S2 Fig — The absolute copy number of FF-Luc mRNA (left) and 18S rRNA (right) from the experiment performed in Fig 2B. Ordinary one-way ANOVA with Dunnett’s multiple comparisons test. ****, p < 0.0001. N=3. For 18S rRNA, no comparisons were statistically significant. (TIF) [file ppat.1012329.s002.tif]

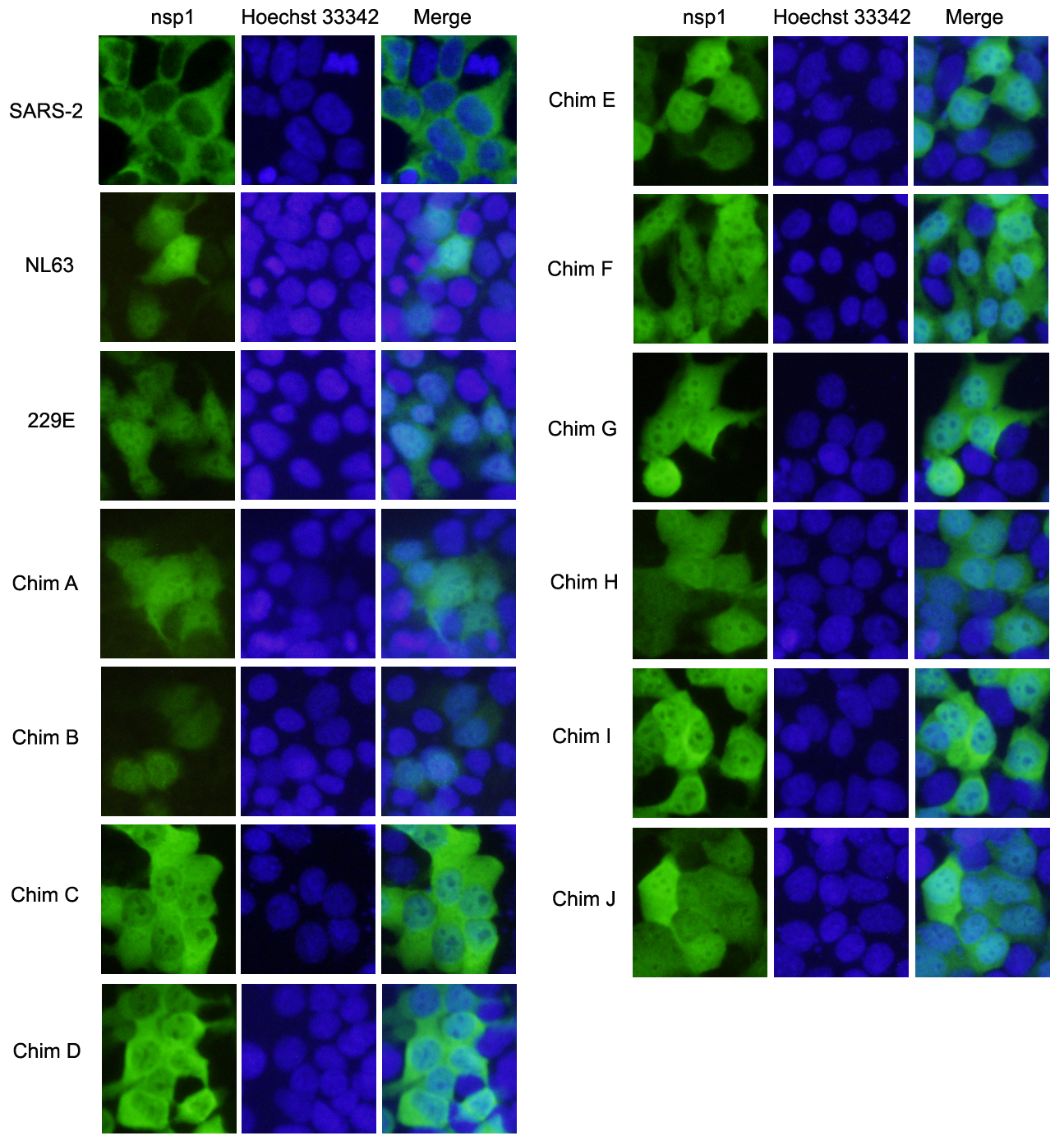

Supplement: S3 Fig — cDNA for chimeric nsp1s described in Fig 3 were transfected into 293T cells and visualized by IF as in Fig 1D. Cell nuclei were counterstained with Hoechst 33342. Images were captured using the 40x objective. (TIF) [file ppat.1012329.s003.tif]

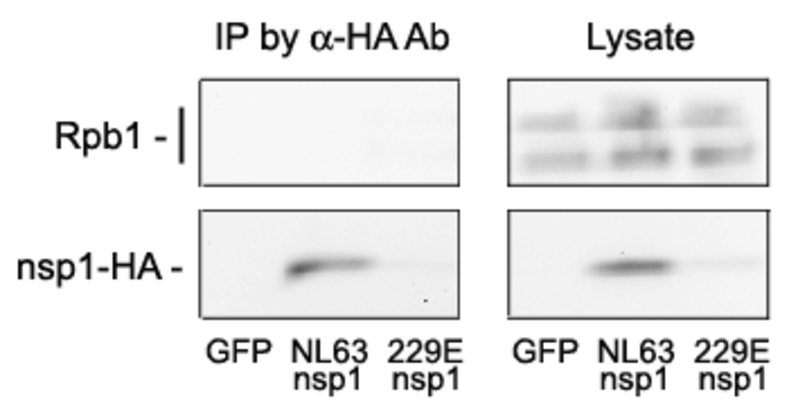

Supplement: S4 Fig — 293T cells pretreated with 5 μM MG132 for 3 hrs were transfected with mRNAs for NL63 or 229E nsp1 or GFP (control). Twenty h after transfection, cell lysates were prepared and applied for immunoprecipitation with anti-HA Ab and analyzed by Western blotting with the indicated Ab. (TIF) [file ppat.1012329.s004.tif]
